# Chromosomal Evolution in Chiroptera

**DOI:** 10.3390/genes8100272

**Published:** 2017-10-13

**Authors:** Cibele G. Sotero-Caio, Robert J. Baker, Marianne Volleth

**Affiliations:** 1 Department of Genetics, Universidade Federal de Pernambuco, Recife 50740-600, Brazil; cibele.caio@gmail.com; 2 Department of Biological Sciences, Texas Tech University, Lubbock, TX 79409, USA; robert.baker@ttu.edu; 3 Department of Human Genetics, Otto-von-Guericke University, Magdeburg 39120, Germany

**Keywords:** bats, chromosomal rearrangements, cytogenomics, karyotype, phylogeny

## Abstract

Chiroptera is the second largest order among mammals, with over 1300 species in 21 extant families. The group is extremely diverse in several aspects of its natural history, including dietary strategies, ecology, behavior and morphology. Bat genomes show ample chromosome diversity (from 2n = 14 to 62). As with other mammalian orders, Chiroptera is characterized by clades with low, moderate and extreme chromosomal change. In this article, we will discuss trends of karyotypic evolution within distinct bat lineages (especially Phyllostomidae, Hipposideridae and Rhinolophidae), focusing on two perspectives: evolution of genome architecture, modes of chromosomal evolution, and the use of chromosome data to resolve taxonomic problems.

## 1. Introduction

Chiroptera includes over 1350 extant species of bats and is the second largest mammalian order, outnumbered only by Rodentia in species diversity [1,2,3,4]. Several particularities characterize the order Chiroptera; the most conspicuous are the morphological attributes associated with the ability of powered flight (e.g., wings, patagia, and rotated hindlimbs) [5]. In addition, other traits associated with flight, such as high metabolic rate and unusual features such as echolocation and longevity make the group unique among mammals [6,7]. Bat species also invaded diverse available niches, displaying a wide range of feeding strategies, such as sanguivory, nectarivory, frugivory, omnivory, and carnivory—including specialized diets, e.g., fish (*Noctilio leporinus*, *Myotis vivesi, Myotis adversus)* [8]; frogs (*Trachops cirrhosus)* [9]; birds (*Nyctalus lasiopterus* [10]); and different kinds of vertebrates (*Megaderma lyra*, *Vampyrum spectrum*, *Chrotopterus auritus*) from an ancestral state of insectivory [11,12,13]. Therefore, morphological, physiological, and behavioral traits are also highly variable and diverse within and among taxa.

The systematic classification of Chiroptera has been the subject of recurrent change. Most reorganization of the bat phylogeny was made after molecular analyses indicated that the historical arrangement of bats into the suborders Micro- and Megachiroptera was not natural (monophyletic). Bat diversity is now subdivided into the suborders Yinpterochiroptera (Pteropodiformes) and Yangochiroptera (Vespertilioniformes), reflecting the closer relationship between the non-echolocating family Pteropodidae (megabats) and six echolocating old-world microbat families [14,15]. In terms of intraordinal arrangements, the number of bat families has also varied considerably, and has risen from 18 to 21, with the recognition of Cistugidae, Miniopteridae, and Rhinonycteridae within the past two decades [16,17,18,19].

Despite the recent reorganization of Chiropteran suborders and families, all bats form a well-supported monophyletic group. Fossil calibrated molecular phylogenies place the origin of the most recent common ancestor of extant bats around 62 million years ago (mya) [3,14,20]. Radiation of families is thought to have occurred early after the divergence of bats from other mammals. Indeed, recent studies suggest that at least 13 extant bat families had already diverged by mid-Eocene [3,20,21,22]. On another note, bats are monophyletic but their sister relationships to other taxa have been controversial. To this date it is not clear what Laurasiatherian orders are closer to Chiroptera (see Nery et al. [23] for a recent review). Even Chiroptera itself was just recognized as part of Laurasiatheria after molecular analyses refuted their placement within the former superorder Archonta [21,24,25].

With the better accessibility to molecular techniques, the advent of whole genome sequencing, and refinement of cytogenetic methods, we are now uncovering additional particularities of the group as a whole as well as of monophyletic lineages within the order (see below). Many unique genomic features have contributed to the extraordinary variation in chiropteran genomic architecture, and now an open field of cytogenomics promises to shed light into how different genomic components converged to shape the extant karyotypes and diversity of bats. In this review, we focus on particularities of bat genomes and how they correlate to recent advances in the study of karyotypic evolution in the group. Trends of chromosomal evolution will be addressed for 12 of all recognized families, focusing on the best-studied Phyllostomidae, Hipposideridae, and Rhinolophidae. Finally, we will discuss the value of cytogenetic studies as a complement to the field of bat systematics and classification.

## 2. Genomic Features of Bats and Perspectives—An Overview of Repetitive DNAs and Implications for Chromosome Evolution

Bat genomes have remarkable features that make them good objects to study the evolution of chromosome architecture. First, bat genome sizes are small and constrained relative to those of other mammalian orders [26,27]. A comparative study of 164 bat species has shown that their average is ~1.5-fold smaller than the average mammalian genome, and within the order genome sizes vary in only 2-fold, whereas a 4-fold variation is reached across mammals [26]. The small genome size of one bat species has been studied in a chromosomal perspective. Kasai et al. [28] measured the guanine-cytosine (GC) content of individual chromosomes of the species *Myotis myotis* and identified GC richness on smaller chromosomes when compared to larger chromosomes. This is a feature shared among bats and birds, but not other mammals so far investigated. They explain their results by loss of AT-rich DNA in small chromosomes, a feature of many types of repeats, including transposable elements.

Because the gene content across mammals is very uniform and conserved, it is a consensus that the small genomes of bats are the result of a reduction of repetitive DNA and not derived from deletions of coding-genes. Unfortunately, few studies have dealt with the characterization of satellite or other repeats in bat genomes. An exception is the recent interest in the transposable element (TE) landscape of vespertilionid bats, which are the only mammals with active DNA transposons (Class II TEs) [29,30,31]. DNA transposons are thought to have ceased their activity at least 40 mya, and are found in mammalian genomes only as fossil (inactive) elements [32]. In vespertilionid bats, however significant accumulation of *Helitrons* and cut-and-paste transposons was detected [29,30,31]. In addition, Miniopteridae, sister to Vespertilionidae, was shown to share some DNA transposon accumulation [33]. In a cytogenetic perspective, data on recent transposon activity is counterintuitive: it is expected that lineages undergoing increased transposon activity would be subjected to higher rates of chromosomal evolution than those that do not [34]. Vespertilionidae, more specifically the genus *Myotis* present unprecedented species diversity (over 100 species [1]), recent transposon activity, variation in number and location of heterochromatic segments and nucleolus organizer regions (NORs), but an otherwise unchanged karyotype with 2n = 44 [35]. Chromosomal mapping of transposons has yet to be performed to reveal whether preferential accumulation of different families of TEs exist along chromosomal regions, but regardless, distribution of these TEs do not seem to be playing a major role in promoting gross chromosomal rearrangements at least in *Myotis* and other genera with the 2n = 44 primitive karyotype.

Investigation of the dynamics of a second class of TEs has revealed interesting trends that can have significant implications for karyotypic evolution in at least two bat families. The retrotransposon Long interspersed element-1 (LINE-1) is a widespread class II TE on mammalian genomes, including bats. Pteropodids are one of few mammal groups for which these TEs have undergone extinction events [36,37,38,39]. Despite the fact that no correlation studies exist, LINE-1 extinctions might have been responsible for the even smaller genomes of the family when compared to other bats [26]. As with class II TEs, no mapping of these elements has been undertaken on pteropodid genomes. The family Phyllostomidae is the only bat group, for which LINE-1 chromosomal distribution has been investigated, also revealing patterns with important implications in a chromosome evolution perspective. In these bats, differential accumulation patterns are found among species [40,41,42], and a massive centromere enrichment of these sequences has been proposed as one of the factors contributing to the genome plasticity of Phyllostomidae in terms of chromosome rearrangements, especially Robertsonian translocations [43].

Until the writing of the present manuscript (August 2017), 14 whole bat genome sequences had been made available, but no chromosome level assembly had been generated. Furthermore, little attention has been given to the repetitive landscape of most species. The landscape of repetitive DNAs in bat genomes can be summarized by studies that mapped few TEs, but mostly ribosomal DNA (rDNA), and telomeric repeats in representatives of diverse bat families [44,45,46,47,48,49,50]. Some of these studies correlated the observed distributional patterns with genomic features. For example, the small size of bat genomes was correlated with the number of chromosome pairs carrying copies of rDNA clusters [45]. In their work Baker et al. [45] propose that the reduced number of ribosomal sites in bats versus other mammals suggest potential mechanisms are in place to ensure the maintenance of a small genome. The restricted distribution/enrichment of the retrotransposon LINE-1 can also serve as a proxy for reduced repetitive DNA in bat genomes [43]. The distribution of telomeric sequences, on the other hand, does not seem as restricted as rDNA or correlated with small genome size in bats. For example, many species present massive amounts of the TTAGGG(n) in pericentromeric regions [41,44,51]. In these cases, it is hypothesized that amplification of telomeric sequences is directly linked with the expansion of satellite families that have incorporated the canonical telomere sequence [52].

Evidence suggests that repetitive DNA can play a significant role in promoting chromosomal rearrangements, meiotic incompatibilities, and thus, isolation and speciation [34,53,54,55]. It is expected then, that organisms with reduced repetitive content and varying degrees of chromosomal reorganization (see below) would allow for a more direct assessment of these correlations. This is particularly valuable because such genomes provide the opportunity to observe the role of changes in the landscape of particular types of repeats without noise from other active repetitive elements. Interestingly, each family has species presenting with increased amount of heterochromatin. Favored locations are the pericentromeric regions (e.g., *Glauconycteris beatrix* [56]) and complete arms consisting solely in repetitive sequences (e.g., *Megaderma lyra* [57]). Therefore, exploring the repetitive landscape is promising to the understanding of the role of non-coding DNA in genome structure and chromosome architecture, as well as in promoting chromosomal rearrangements in bats.

## 3. Evolution of Genome Architecture

Chromosomal variation has been hypothesized to play a role in turning bats into the second most diverse order of mammals [58]. Nevertheless, most of what we know about “comparative genomics” and chromosomal evolution of Chiroptera derives from classical cytogenetics, especially comparative G-banding studies, as well as the most recent (but limited to a few taxa) data on chromosome painting. So far, chromosome painting has been used to unravel the regions of homology in 69 bat species and was particularly useful when comparing species that have undergone extreme karyotype reshuffling [41,59,60,61].

Through combination of G-banding and chromosome painting, Volleth et al. [62] have shown that bat genomes consist of 25 evolutionary conserved units (ECUs), which are chromosomal blocks conserved in most investigated species. The chromosomal evolution of bats seems to have been shaped mainly by the reshuffling of ECUs in a smaller or higher degree, depending on the mode of chromosomal evolution of particular lineages. Nevertheless, a few groups have undergone extreme karyotype reshuffling, which included ECU disruptions, inversions and other rearrangements. As with other mammals, the trends of karyotypic evolution vary within and among families. Below, we describe the three modes of chromosomal evolution proposed by Baker and Bickham [63], with updated examples and comments.

### 3.1. Conserved Karyotype Evolution

Most bat families are regarded as extremely conserved concerning variation in diploid chromosome number. To generalize Chiroptera as a karyotypically conserved group is an overstatement, since the ancestral (and modal) karyotypes are usually very distinct between families. Similarly, families regarded as extremely conserved, are usually those with the lowest species diversity. Therefore, chromosome variation in lineages within bat families can range from conserved to highly variable and correlate with diversity of groups. Good contrasting examples are Noctilionidae and Vespertilionidae. The former is represented by only two species that maintained their proposed ancestral family karyotype [63]. Vespertilionid bats, with the largest species diversity within Chiroptera (over 400 species) are remarkable for the number of speciation events without chromosomal change in some groups. Namely, the genus *Myotis*, currently has ~100 recognized species, all with an invariable 2n = 44, FN = 52 karyotype. This genus is ~30 million years old and distributed worldwide, including remote islands and on both sides of well-established geographical barriers. Despite all possibilities for geographic and genetic isolation, karyotypic reshuffling has not been part of diversification processes of these bats. Transposon activity is a unique feature of vespertilionid bats and has been hypothesized as the booster for speciation through differential patterns of gene expression [64]. Other vespertilionid lineages, however have reshuffled the ancestral vespertilionid karyotype [65,66], which is somewhat expected for a very speciose and cosmopolitan family.

### 3.2. Moderate Chromosomal Evolution

As with most mammals, some bat lineages present karyotypic diversity at some degree. Firstly, although families can have a conservative nature of karyotypic evolution, most have distinct ancestral karyotypes. This is evidence of at least a moderate karyotype reshuffling specific to each family. Baker and Bickham [63] have proposed the term “karyotypic orthoselection” to describe the cases of moderate chromosomal evolution in bat lineages, in which considerable number of only a few types of rearrangements occurred. The most common rearrangements in bat lineages that underwent karyotypic orthoselection are Robertsonian translocations, resulting in unchanged G-banding pattern of chromosomal arms among taxa.

### 3.3. Extreme Karyotype Reshuffling

Bats have been used as models to describe events of unprecedented genome reshuffling. Karyotypic megaevolution, characterized by fixation of a high number of rearrangements, cannot be explained by any model of chromosomal evolution to date, but seems to have played a major role in the chromosomal evolution of some lineages [63]. Good examples are the phyllostomid lineages giving rise to the genus *Tonatia* (Phyllostominae), to different species in the genus *Micronycteris* (Micronycterinae), as well as to the vespertilionid *Lasionycteris noctivagans*. As a result of extreme karyotype reshuffling, homologous chromosomal segments of the species in question and their ancestral karyotypes can only be determined by fluorescence in situ hybridization (FISH) and not by G-band patterns. Indeed, despite successful homology detection, chromosome painting in *Tonatia saurophila* [41,61] and *Micronycteris hirsuta* [60] could not determine the order of rearrangements that took place for the fixation of their karyotypes from closely related species.

## 4. Overview of Chromosomal Evolution within Chiropteran Families

Closer examination of the chromosomal evolution in Chiroptera shows that each family has its characteristic mode of chromosomal changes. For 12 families, the current knowledge is summarized below. For another 9 small families the data can be taken from Table 1.

### 4.1. Yinpterochiroptera (Pteropodiformes)

Yinpterochiroptera [107] or Pteropodiformes [108] currently comprise the megabats (family Pteropodidae), plus 6 families, that are grouped together in the superfamily *Rhinolophoidea*: Rhinolophidae, Hipposideridae, Rhinonycteridae, Rhinopomatidae, Megadermatidae, and Craseonycteridae [3,14,109].

#### 4.1.1. Pteropodidae

The flying foxes or Pteropodidae show diploid numbers ranging from 2n = 24 to 58. However, most of the species investigated cytogenetically possess one of three modal chromosome numbers (2n = 34, 36, or 38), and to this date, only 8 of the 45 karyotyped species were shown to have a divergent 2n. In total, 13 species have been studied with G-banding. FISH analyses were undertaken for 5 species from 4 genera (2n in parentheses), i.e., *Cynopterus* (34), *Eonycteris* (36), *Rousettus* (36), and *Pteropus* (38) [62,68,74,75,97].

**Figure 1:**
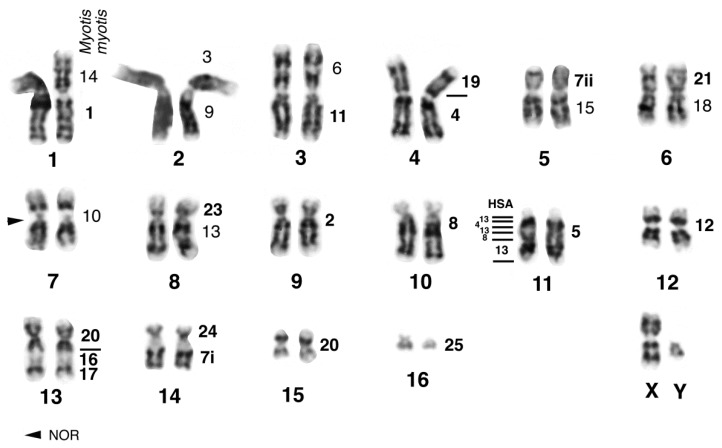
G-banded karyotype of the pteropodid *Macroglossus sobrinus*, 2n = 34, FN = 60 (Senckenberg Museum Frankfurt accession number SMF 87472), from Ulu Gombak, Malaysia. The numbers on the right side of each chromosomal pair indicate homology to the vespertilionid bat *Myotis myotis* revealed by FISH (bold numbers) or G-band comparison with the karyotype of *Eonycteris spelaea* (Pteropodidae). The numbers on the left side of pair 11 indicate homology to human chromosomes 4, 8 and 13. In *Macroglossus*, the order of these segments is changed compared to that in *Eonycteris* (13-4-8-13). The arrowhead points to the position of the nucleolus organizer regions (NOR). Applied methods are described in literature [62,110,112].

Before giving an overview on the chromosomal evolution of flying foxes, we will present new original data on a species belonging to the subfamily Macroglossinae. Herein we describe the karyotype of *Macroglossus sobrinus* based on G-bands and FISH with *M. myotis* whole chromosome paints (supplemented by human, tree shrew and *Eulemur* paints; see the methods in Volleth et al. [110] for reference). Nectar-feeding species belonging to the genus *Macroglossus* are distributed from India to Australia. Due to similar feeding strategies, they were formerly grouped together with *Eonycteris* in the subfamily Macroglossinae. However, detailed morphological studies [111] and DNA sequence analyses revealed that both genera are in fact distantly related [3]. In contrast, the molecular hypothesis of a close relationship between *Eonycteris* and *Rousettus* (subfamily Rousettinae) is also supported by karyotype similarities [97]. The karyotype of *M. sobrinus*, based on the analysis of one male and one female from Peninsular Malaysia (Ulu Gombak, Selangor), consists of 2n = 34 and FN = 60. All autosomes were bi-armed, however the short arm of the smallest pair consisted of heterochromatin and was therefore not counted for the FN. The X chromosome is a medium-sized metacentric and the largely heterochromatic Y chromosome is a small metacentric. Heterochromatin was found only at the centromeres. The secondary constriction close to the centromere in the short arm of *M. sobrinus* chromosome MSO7 was detected as the single NOR site by silver-staining. Homology to *Myotis* is indicated on the G-banded karyogram in Figure 1.

The composition of 13 autosomal pairs is similar in *Eonycteris*, *Rousettus, Pteropus*, and *Macroglossus*. However, structural differences were found in the elements homologous to *Myotis* chromosomes (MMY) 16/17, 20, 24, and the element homologous to the proximal region of MMY7 (named 7i). In *Pteropus*, which displays the highest chromosome number of the mentioned genera (2n = 38), these elements represent individual chromosomes, partly altered by terminal heterochromatin additions [97]. The 2n reduction from 38 to 34 in *Macroglossus*, unlike in *Eonycteris* and *Rousettus*, is not the result of a terminal fusion of the MMY homologs 24 and 16/17 [62,75,97]. Instead, the 2n reduction in *Macroglossus* is the result of centric fusions of MMY24 and MMY7i on one hand (MSO14) and a tandem fusion of MMY20 and MMY16/17 homologous chromosomes (MSO13). Further, the ancestral acrocentric shape of MMY7i has been changed in *Eonycteris* and *Rousettus*, resulting in a small metacentric chromosome. As a consequence, the fusion chromosome 24-16/17 found in *Eonycteris* and *Rousettus* has to be considered as derived and as an independent event from a similar fusion in *Hipposideros* [97].

The karyotype of *Pteropus* has further been shaped by a pericentric inversion in the metacentric chromosome homologous to MMY19/4 [97]. The chromosomal arms composing the 11 fusion chromosomes in *Cynopterus* differ completely from those of the abovementioned genera. Further, the ancestral bi-armed elements homologous to MMY10 and MMY12 were changed by a centric fission and MMY20 by an inversion, and subsequent centric fusion with the formerly short arm of the MMY10 homolog (10i). Therefore, the impressive karyological differences between *Cynopterus* and the other pteropodids mirror the molecular results which assign *Cynopterus* to the first branch on the pteropodid tree [3].

A large comparative banding study including 8 African pteropodid species with 2n between 34 and 36 was undertaken by Haiduk et al. [113]. In addition to extensive heterochromatin additions in some species, several indications for chromosomal rearrangements were found leading the authors presume “a substantially higher rate of chromosomal change than suggested by non-differentially stained karyotypes”.

Y chromosomal rearrangements have been postulated in African pteropodids where the males were found to have a 2n = 35, but the females a 2n = 36. This group however, needs a closer examination. Only for *Micropteropus pusillus* a differentially stained karyotype is available [113]. For *Epomops buettikoferi*, *Epomophorus crypturus* and *Epomophorus gambianus* only non-differentially stained chromosomes were published [89,114].

From the above-mentioned examples, it becomes clear that chromosomal analyses with application of painting probes are highly desirable to unravel the chromosomal evolution in the family Pteropodidae. In flying foxes, instead of Robertsonian translocations other chromosomal rearrangements as inversions and tandem fusions are likely to have played a role in chromosomal evolution. For detection of such changes application of modern molecular cytogenetic techniques is necessary.

##### Rhinolophoidea

The family Megadermatidae, together with Rhinopomatidae, Craseonycteridae, Rhinonycteridae, Hipposideridae, and Rhinolophidae, constitutes the superfamily Rhinolophoidea within Yinpterochiroptera [3].

#### 4.1.2. Megadermatidae

Megadermatidae comprises only six species arranged in five genera [115], and despite the low number of species, the 2n and G-banded karyotypes are known only for *Megaderma* (*Lyroderma) lyra* (2n = 54) [57] and *M. spasma* (2n = 38 or 2n = 46) [68,78,116]. The chromosome number of specimens from the Philippines differs from those found in other regions, suggesting that *M. spasma* may include cryptic species [116]. Chromosome painting with human probes was applied on a Chinese specimen of *M. spasma* (2n = 38) to define chromosome homologies between karyotypes of the two species [68]. Further comparison with *Myotis* karyotype showed that six ECUs are disrupted in *M. spasma* karyotype (Volleth et al., own unpublished results). Overall, the distinct chromosome numbers, as well as intraspecific variation detected, indicate that a large number of rearrangements must have occurred in *Megaderma* since its divergence from the remainder Rhinolophoidea families.

#### 4.1.3. Hipposideridae

Cytogenetic studies were undertaken in 6 of the 8 hipposiderid genera, thus the chromosomal numbers for *Anthops* and *Paracoelops* are yet unknown. More statistical details can be found in Table 1. For a closer insight into the karyotypic evolution of this family, we will first describe the karyotype of the diadem roundleaf bat, *Hipposideros diadema*.

**Figure 2:**
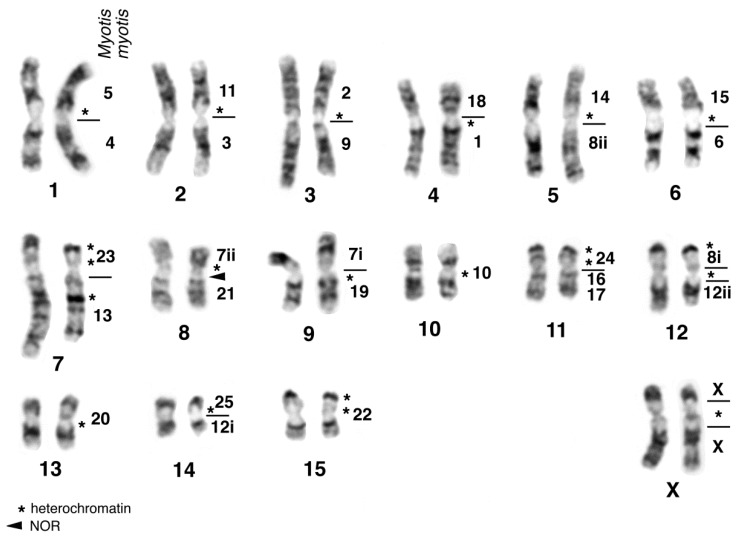
G-banded karyotype of *Hipposideros diadema*, 2n = 32, FN = 69 (SMF 69296) from Ulu Gombak, Malaysia. The numbers on the right side of each chromosomal pair indicate homology to *M. myotis*. The suffix i or ii refers to proximal or distal segments of those *Myotis* chromosomes which are present as two entities in the *Hipposideros* karyotype. The arrowhead points to the position of the NOR and the asterisks indicate heterochromatic segments. For methods, see Figure 1.

As with the majority of species of the genus *Hipposideros*, the karyotype of *H. diadema* (HDI) shows a diploid chromosomal number of 2n = 32 with a FN = 60. Up to now, only homogenously stained karyotypes had been published from Bornean and Philippine specimens [116,117]. The karyotype of the female studied by us consisted of 15 bi-armed autosomal pairs and large submetacentric X-chromosomes. The composition of the autosomal pairs, revealed by G-banding and fluorescence in situ hybridization with MMY painting probes, complemented with some human probes was found to be similar to that of other 2n = 32 *Hipposideros* species [62,75,76]. Ag-NOR staining revealed a single rDNA site on the long arm of pair HDI8 close to the centromere. Analysis of 13 metaphase plates resulted in a mean value of 1.9 NORs per cell. Chromosomal arm HDI8q is homologous to MMY21 and therefore the location of the NOR in *H. diadema* is the same as found in other hipposiderids and rhinolophids. C-banding revealed enlarged centromeric heterochromatic regions and interstitial and telomeric heterochromatic segments on several chromosomes. Fluorescence staining with the dyes CMA (chromomycin A3) and DAPI (4′-6-diamino-2-phenylindole) resulted in enhanced fluorescence of the centromeric heterochromatin by CMA in all autosomal pairs except HDI7, where the centromeric heterochromatin was Distamycin A/DAPI (DIDA) positive. The large centromeric block on the X chromosomes was CMA and DIDA positive. The terminal heterochromatic segments on the short arms of pairs HDI11, 12, 15 and the interstitial segment in the long arm of pair HDI7 were shown to be DAPI positive. The heterochromatic segments and homology of chromosomal arms to the vespertilionid *M. myotis* are indicated on the G-banded karyotype of HDI in Figure 2.

The amount and location of C-positive heterochromatin varies among *Hipposideros* species [76,77]. In addition to *H. diadema*, also *H. larvatus*, *H. armiger* and *H. pratti* show interstitial and terminal C-blocks, whereas heterochromatin is restricted to centromeric regions in *H. pomona* [76]. This finding coincides with molecular data which revealed a closer relationship of the four above mentioned species compared to the distantly related *H. pomona* [3,118].

In addition to the above mentioned *Hipposideros* species, results of G-banding and FISH are available for *Aselliscus stoliczkanus* (2n = 30) [74,75]. A comparison of the arm combinations between *Aselliscus* and *Hipposideros* revealed two possible synapomorphic Robertsonian fusion combinations, i.e., homologs to MMY14/8ii and 19/7i [112]. Therefore, from the 14 autosomal pairs of *Aselliscus*, three represent ancestral elements, two are shared with the genus *Hipposideros*, and 9 are apomorphic combinations. Especially noteworthy is the single-armed condition of the originally bi-armed elements homologous to *Myotis* chromosomes 20 and 22 in *A. stoliczkanus*.

Only few species of *Hipposideros* have been reported with a deviating diploid number. Among them are *H. obscurus* from the Philippines (2n = 24) [116] and the African species *Doryrhina cyclops* (2n = 36) [119], removed from *Hipposideros* for morphological and molecular reasons by Foley et al. [118]. Only homogeneously stained karyotypes are available from these species. A detailed study was undertaken for *Macronycteris commersonii* from Madagascar with 2n = 52 and an acrocentric dominated karyotype [97]; also removed from *Hipposideros* for morphological and molecular reasons by Foley et al. [118]. The closely related species *M. gigas* and *M. vittatus* have also been reported to have a 2n = 52 (only by non-differentially stained karyotypes; for citations see Richards et al. [97]).

In *M. commersonii*, the composition of the 5 bi-armed pairs was revealed by FISH with *Myotis* paints and showed the presence of 4 ancestral elements (homologous to MMY10, 20, 22 and the Robertsonian fusion product MMY23/13), which were found also in Pteropodidae [62,75,97]. The largest bi-armed autosomal pair of *M. commersonii*, however, is composed of arms homologous to MMY3 and 11 [97], a combination otherwise only found in the genus *Hipposideros*. Together with the common fission of the plesiomorphic bi-armed homolog to MMY12, this arm combination could therefore be an indication for a closer relationship of *M. commersonii* to the genus *Hipposideros*. Such relationship was supported by some molecular data, but rejected by others as molecular phylogenies have resulted in divergent topologies for Hipposideridae [3,19,20,118]. Therefore, results of molecular-cytogenetic investigations in the genus *Asellia* and *Doryrhina* are needed for a better insight of the chromosomal evolution of this family.

In spite of the large-scale conservation of chromosomal arms in the genus *Hipposideros*, a structural change in the short arm of the ancestral element homologous to MMY23/13 could be traced using human whole chromosome painting probes for chromosomes 11, 12 and 22. In contrast to the ancestral pattern 11-22-12 (from centromere to telomere) present in the pteropodid *Eonycteris* and the hipposiderids *M. commersoni* and *Aselliscus* [62,75], and Volleth own unpublished data, the sequence 22-12-11 was found in *H. diadema* and *H. larvatus* together with an altered G-banding pattern. A similar rearrangement was proposed for *H. armiger* and *H. pratti* [76]. Based only on differences in the G-banding pattern, Mao et al. [76] suggested a paracentric inversion in this chromosome in *H. pomona*. It should be noted that the G-banding pattern of this chromosome is influenced by the presence or absence of terminal heterochromatic segments. Additional *Hipposideros* species need to be studied with human paints to decide if this feature can be used to define intrageneric relationships. In summary, from data obtained thus far, the mode of karyotypic evolution in the family Hipposideridae can be considered as moderate.

#### 4.1.4. Rhinolophidae

The monotypic family Rhinolophidae (horseshoe bats) is relatively well studied. Diploid numbers are known for 46 of the 96 currently recognized species [102]. G-banding and FISH was applied on 20 and 13 species, respectively. The distribution of chromosomal numbers coincides with the geographical distribution of the two main clades i.e., the Afro-Palearctic clade with 2n = 58 and the Asian clade with 2n = 62. Both clades were resolved also in most molecular-based phylogenies [120,121]. Of the 18 karyologically-studied species within the Afro-Palearctic clade, the species *Rhinolophus hipposideros* is peculiar because of three geographically separated chromosomal variants with diploid numbers of 54, 56 and 58 [98,99]. In addition to currently known 13 species with 62 chromosomes (Asian clade), a high diversity of chromosomal numbers was identified in Asian rhinolophids. A morphologically clearly defined group is the *R. trifoliatus* clade with 2n between 28 (*R. sedulus*) and 52 (*R. formosae*) and extremely high rates of chromosomal changes which led to the detection of cryptic species in the “*R. luctus* complex” [100,101,102]. The second group of Asian *Rhinolophus* species with chromosomal numbers from 36 to 56 consists of *R. pearsoni*, *R. rouxi* and related species. However, for an unequivocal identification of chromosomal changes, analysis of the respective karyotype with molecular cytogenetic methods is needed because of similarity in G-banding patterns of the numerous small chromosomal arms. Such experiments were done in *R. pearsoni pearsoni* (2n = 44; but not in *R. p. chinensis* with 2n = 42), *R. sinicus* (2n = 36) [75,79], and *R. rouxi* (G-bands published by Zima et al. [122], fusion chromosomes given in Table 2). In spite of the quite high number of studied species, the course of chromosomal evolution from a presumed rhinolophid ancestor to the evolutionarily stable karyotypes with 2n = 58 or 62 remains speculative. The following features can be taken into consideration for evaluation of an ancestral karyotype. First, the search for preservation of ancestral chiropteran chromosomes (ECUs) resulted in two elements: small bi-armed chromosomes homologous to MMY20 and 22. These are preserved only in species of the Afro-Palearctic clade but found separated in two chromosomal arms (and partially fused with other arms) in all other *Rhinolophus* species. The bi-armed homologs to MMY10 and MMY12, which are present in the Pteropodidae and in the hipposiderid *Aselliscus*, are found separated into two elements each in all rhinolophids. Further, comparison of arm combinations of Robertsonian fusion chromosomes found in recent species with those present in out-group taxa could give valuable hints for the search of the ancestral rhinolophid karyotype. However, as Robertsonian translocation is the prevalent mode of chiropteran chromosomal evolution [68], similarities in arm combination should be considered with caution. Table 2 lists all reported compositions of fusion chromosomes in Rhinolophidae (9 species) and those of some outgroup species (*Aselliscus*, *Hipposideros* and *Eonycteris*). Remarkably, 63 different arm combinations were found in these 9 species. The species presenting the highest number of common fusions with an out-group taxon was *R. p. pearsoni.* It shows two fusions in common with *Aselliscus* (1/16-17 and 3/15) and one common combination with the genus *Hipposideros* (4/5, present also in *R. hipposideros* and *R. sedulus*).

The different positions of *R. hipposideros* in molecular-based phylogenies, i.e., either as basal branch of the Afro-Palaearctic clade or sister to the basal *R. trifoliatus* clade, can be explained by use of different data sets. Analyses of mitochondrial DNA (mtDNA) resulted in a relationship to the *R. trifoliatus* clade [121,123], whereas nuclear data sets revealed a close relationship to the Afro-Palaearctic clade [121]. Cytogenetic data clearly indicate a close relationship of *R. hipposideros* to the Afro-Palaearctic clade. Additionally, the retention of a presumed ancestral fusion chromosome supports a basal position of this species in the rhinolopid phylogeny.

In summary, chromosome studies in Rhinolophidae revealed that speciation occurred without detectable chromosomal changes in the 2n = 58 and the 2n = 62 karyotypes of the Afro-Palearctic and Asian clade, respectively. In contrast, extreme karyotype reshuffling in the low-2n species of the *R. trifoliatus* and *R. rouxi* clades may have been related to speciation.

### 4.2. Vespertilioniformes or Yangochiroptera (sensu Teeling et al., 2002)

This suborder is composed of three superfamilies, Noctilionoidea, Emballonuroidea and Vespertilionoidea. Six families are included in the superfamily Noctilionoidea: Mystacinidae, Furipteridae, Noctilionidae, Thyropteridae, Mormoopidae, and Phyllostomidae [3], but only Mormoopidae, Noctilionidae, and Phyllostomidae were better investigated cytogenetically. The superfamily Emballonuroidea includes the families Emballonuridae and Nycteridae, for which no comprehensive cytogenetic studies were undertaken, but available data will be discussed below. Finally, in addition to the large families Molossidae and Vespertilionidae, the superfamily Vespertilionoidea is composed of the Old World Miniopteridae and the neotropical Natalidae. The fifth family, Cistugidae, comprises only two species formerly assigned to the genus *Myotis*. We discuss the karyotypic trends of the first three mentioned Vespertilionoidea families.

#### 4.2.1. Mormoopidae

The family Mormoopidae comprises two genera, *Pteronotus* and *Mormoops*, with eleven currently recognized species [2]. There is however, recent molecular evidence of species-complexes in *Pteronotus*, which alone would raise the total number of mormoopid species to 15 [124,125]. Efforts to cytogenetically characterize Mormoopidae species are restricted to few studies which however, cover the two *Mormoops* and six *Pteronotus* species historically recognized: *M. blainvillei*, *M. megalophylla*, *P. parnellii*, *P. personatus*, *P. macleayii*, *P. quadridens*, *P. gymnotus*, and *P. davyi* [82,83]. G-banding analyses indicated that the karyotypic evolution of Mormoopidae is very conservative, with a 2n = 38 and FN = 60 in all species [63]. G-banding patterns are identical in all *Pteronotus* species investigated and differ from *Mormoops* G-bands in a single chromosome (second largest arm). Furthermore, a second chromosome arm is slightly different and unique to *M. blainvillei* [82]. The increased reports of diversity in *Pteronotus* was not followed by the same amount of cytogenetic studies. Most of karyotypic descriptions and available G-bands come from a few localities and not the whole range of the genus. Therefore, despite presenting a conservative mode of chromosomal evolution, chromosomal studies of newly described species might still uncover unknown karyotypic variation in the genus.

#### 4.2.2. Noctilionidae

Like Mormoopidae, the family Noctilionidae is neither speciose, nor karyotypically diverse (see “Conservative karyotypic evolution” section above). This monotypic family has only two recognized species: *Noctilio leporinus* and *N. albiventris* [1]. Despite a recent suggestion that *N. albiventris* might harbour cryptic diversity [126], the use of multiple molecular datasets indicated that different patterns of gene flow of genetic transmission elements are consistent with the current subspecies delimitation of the genus [127]. Interestingly, the two species of *Noctilio* are hypothesized to have almost identical karyotypes (2n = 34; FN = 62) to the karyotypes of mormoopids [83]. Individuals from multiple localities across *Noctilio* range were investigated with C- and G-banding, as well as Ag-NOR staining [83,87,88]. In situ hybridizations using *Macrotus californicus* (Phyllostomidae) probes have confirmed the proposed similarities among *Noctilio* and *Pteronotus*, which differ by two Robertsonian translocations and short-arm heterochromatic additions on two *Noctilio* chromosomes (Sotero-Caio et al., own unpublished data).

#### 4.2.3. Phyllostomidae

**Figure 3:**
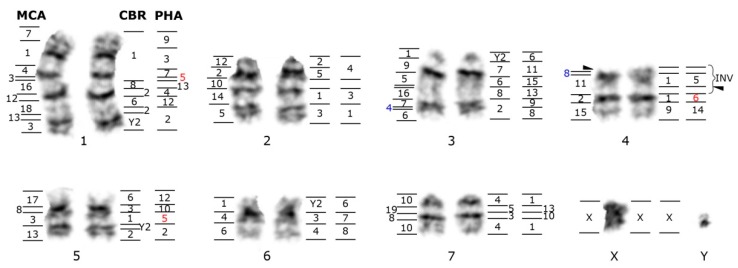
G-banded karyotype of the phyllostomid *Tonatia saurophila* (TSA), 2n = 16, FN = 22 from Ecuador (Museum of Texas Tech accession number TK104519) showing the magnitude of chromosomal rearrangements when compared to the karyotypes of other three phyllostomid bats. The numbers on the left side of each chromosomal pair indicate homology to *Macrotus californicus* (MCA) from Sotero-Caio et al. [41]; the numbers on the right indicate homologies detected in a TSA specimen from Brazil to the chromosomes of *Carollia brevicauda* (CBR) and *Phyllostomus hastatus* (PHA) by Ribas et al. [61]. Note that the specimens analyzed differ by one pericentric inversion on pair 4. Numbers in blue correspond to small chromosome fragments detected with MCA, but not with CBR and PHA painting probes. Numbers in red are being investigated to confirm homology assignments (Yang and Fu, personal communication). Arrowheads indicate the sites of 45S ribosomal DNA cistrons.

Phyllostomidae is by far one of the best genetically investigated families and this is particularly true from a cytogenetic perspective. Diploid numbers are known for approximately one third of the 204 recognized species [2] and vary considerably from 2n = 14 to 2n = 46 (Table 1). In addition to the multiple studies describing C and G-banded karyotypes, chromosome homology of 25 species has been investigated through chromosome painting [41,59,60,61,92,93,94,95,96], and whole sex chromosome probes have been applied to meiotic chromosomes of four species [128,129]. Sets of whole chromosome paints have been produced from three species to aid in comparative studies: *Phylostomus hastatus*, *Carollia brevicauda*, and *Macrotus californicus* [59,96]. The proposed ancestral karyotype for the family has a 2n = 46, FN = 60, and only the species *Macrotus waterhousii* has retained this putative ancestral state [83,130]. Most of the subfamilies on the other hand, have a highly derived karyotype when compared to the genus *Macrotus* [63]. Interestingly, different phyllostomid lineages have varying karyotypic evolution trends, including conserved, moderate, and intense levels of chromosome reshuffling. A striking example of chromosome reorganization is the phyllostomine *Tonatia saurophila* with a 2n = 16, and FN = 20 [61] or 22 [41]. At least 26 unique rearrangements were fixed after radiation of *Tonatia* from other members of the subfamily Phyllostominae, which in turn present a conservative trend of chromosomal evolution [41]. Integration of hybridization results using chromosome painting with probes from three phyllostomid species on *Tonatia* karyotype illustrates how syntenic association differ in these species (Figure 3). Other examples of considerable divergence from the family ancestral karyotypes detected by chromosome painting are *C. brevicauda* (Carolliinae), *Anoura cultrata* (Glossophaginae), *Uroderma bilobatum* and *U. magnirostrum* (Stenodermatinae), and the micronycterine *M. hirsuta* [59,60,94,96]. Despite the high degree of syntenic association variation in Phyllostomid bats, synapomorphic segment associations have been found in groups of species within the same subfamily: examples are at least six and three shared chromosome characters between the vampire bats (Desmodontinae), and the fruit eating phyllostomids (Stenodermatinae) [61,93,95].

G-banding and chromosome painting were also helpful in revealing that sex-autosome translocations are relatively common in phyllostomid bats. However, these rearrangements are not randomly distributed across the group and restricted to clades within the subfamilies Glossophaginae, Carolliinae, and Stenodermatinae. Therefore, although the number of species with multiple sex systems is high, they only evolved three times independently in the family and were retained after speciation events. Indeed, all species within the speciose subfamily Stenodermatinae (roughly 1/3 of Phyllostomidae diversity), share a synapomorphic X-autosome translocation, in which the X-translocated autosome is homologous to PHA15. In this case, species have a multiple sex system with XX females and XY_1_Y_2_ in males, where the Y_2_ corresponds to PHA15, and males have uneven diploid numbers. In addition, other sex systems derived from this translocation have been reported, such as Neo-XY systems, where the free homolog of the autosome (Y_2_, PHA15) is translocated to the Y_1_ chromosome, resulting in females and males with the same 2n (e.g., *Uroderma*). Two unique examples derived from the Neo-XY system are found in *Mesophylla macconnelli* with translocation of a second autosome to the Neo-Y, resulting in a Neo-X_1_X_2_Y system in males and Neo-X_1_X_1_X_2_X_2_ in females, as well as the secondary fission of the Neo-X in *Vampyressa thyone*, which resulted in a X_1_X_2_Y/X_1_X_1_X_2_X_2_ in males and females, respectively. In both species, males have one less chromosome than females, with 2n = 21/22 and 23/24, for *M. macconnelli* and *V. thyone*, respectively [93]. Other sex-autosome translocations that occurred independently in Phyllostomidae include the X-PHA5/2 in *Carollia brevicauda*, probably shared with other seven *Carollia* species, and lost in a single population of *C. castanea* [59,131,132]; and the X-unidentified autosome in *Choeronyscus godmani* (Glossophaginae) [133]. Changes in the sex chromosome system are extremely rare events in Eutheria. In Chiroptera, only few other non-phyllostomid bats have multiple sex chromosome systems. Examples are the pteropodid *Micropteropus pusillus* (suspected also in closely related species) [113], some species out of the *Rhinolophus trifoliatus* clade [101], *Myzopoda aurita* [80] and the vespertilionids *Glischropus tylopus* [66] and *Glauconycteris beatrix* [56].

#### 4.2.4. Nycteridae

This small insectivorous Old World family comprises only a single the genus, *Nycteris*, consisting of 14 African and 2 Asian species. Only non-differentially stained karyotypes from 6 African species have been published up to now (Denys et al. [89] and citations therein). Three different diploid numbers ranging from 2n = 34 to 2n = 42 were reported. The majority of species (4) show a 2n of 42 [89]. Deviating chromosomal complements were reported for *N. intermedia* (2n = 34) and *N. macrotis* (2n = 40). For judging the extent of chromosomal rearrangements, however, differentially stained karyotypes, preferably supplemented with FISH analyses, would be needed to determine the mode of chromosome evolution in the family.

#### 4.2.5. Emballonuridae

Emballonurids are considered the most primitive representatives of the suborder Vespertilioniformes and comprise two subfamilies, Emballonurinae and Taphozoinae. Cytogenetic studies of Emballonurinae revealed certain variation in diploid numbers, with a single known diploid number (2n = 24) in representatives of the Old World genus *Emballonura* [78,117], variations in 2n from 22 to 32 in 7 neotropical genera [69,86], and 2n = 48 for the genus *Peronymus* [73]. In spite of a relatively small range of diploid numbers, karyotypic comparison in Diclidurini (neotropical emballonurids) by G-bands revealed extensive chromosomal divergence [69]. The second subfamily Taphozoinae comprises only two genera with diploid numbers of 2n = 42 and 44, for *Taphozous* and *Saccolaimus*, respectively [69,72]. G-banded karyotypes are known all together from 10 species of emballonurids [68,69,71]. *T. melanopogon* is the only species where FISH using human painting probes has been applied [68]. An impressive observation was recently made in *Cormura brevirostris* (2n = 22) from Brazil. In this species, 8 autosomes showing only monobrachial homology and the presence of a multivalent ring in male meiosis [70] may indicate chromosomal evolution by multiple simultaneous whole arm translocation events, a novelty for Eutheria. Based on their wide range of chromosomal numbers and divergences in the G-banding pattern, emballonurids can be considered a group with considerable amounts of karyotypic evolution.

#### 4.2.6. Molossidae

Conventionally stained karyotypes have been investigated for about 50% of the species of free-tailed bats or Molossidae. In the majority of species (41), a diploid chromosome number of 48 was found. A deviating 2n in the wide range from 34 to 52 was found in only 9 species (for references see Sreepada et al. [134]). G-banded karyotypes were studied from 11 [135,136,137] and FISH analyses were done on 3 species [62,68,80]. These studies confirmed the conservation of chromosomal arms in Molossidae.

Remarkably, despite constancy in 2n, the number of autosomal arms, FN, was found to vary from 54 to 66 in the 2n = 48 species. In Molossidae, the FN is difficult to determine as several pairs might have minute short arms and thus a subtelocentric shape. However, the detection of these short arms depends on the quality of chromosome preparation and the level of condensation [137] and might even be more difficult to ascertain in G-banded chromosomes due to the swelling process. Nevertheless, these differences are important as they mirror the mode of chromosomal evolution in this family which seems to be based mainly on small pericentric inversions. Additionally, also variation in number and location of NORs has played a role in the chromosomal evolution of molossids [137]. Although the chromosomal evolution in Molossidae is generally characterized by intra- and intergeneric conservativism, intrageneric and partly also intraspecific variation was detected in the genera *Molossops* and *Eumops* [137,138,139].

#### 4.2.7. Miniopteridae

Formerly belonging to the family Vespertilionidae, the genus *Miniopterus* has finally been assigned to a separate family, Miniopteridae. This decision was based on results of mtDNA analysis and long-known apomorphic morphological features (Hoofer and van den Bussche [17] and citations therein). The diploid number is known from altogether 10 species and it is invariably 46. Thus, the mode of chromosomal evolution within the family Miniopteridae is conservative [140]. G-bands have been published for three species, *Miniopterus schreibersi*, *M. fuliginosus* and *M. griveaudi*, and FISH data using *Myotis* probes were also reported for the last two species [79,80,81]. Compared to *Myotis*, whose karyotype comes close to the ancestral vespertilionid karyotype [141], the following differences were stated: (i) two acrocentric chromosomes were found instead of the metacentric *Myotis* chromosome MMY3/4, this is very likely the plesiomorphic condition; (ii) the MMY12 homologous element is bi-armed with a G-banding pattern similar to the ancestral state; (iii) chromosomes homologous to *Myotis* MMY7 and MMY10 display an apomorphic G-banding pattern found nowhere else up to now; and (iv) the chromosome homologous to the metacentric MMY16/17 shows an acrocentric shape [79]. In the Miniopteridae karyotype no feature which could serve as synapomorphy indicating a closer relationship to any other vespertilionoid family has been found.

#### 4.2.8. Vespertilionidae

As the neotropical Phyllostomidae, the world-wide distributed family Vespertilionidae is cytogenetically well studied, partly due to its distribution not only in tropical, but also in temperate climates. The diploid numbers ranging from 18 to 52 are known for 194 species. 76 species were G-banded and FISH was applied onto 12 species [62,65,66,75,79,80,106,142]. The most common diploid number of 2n = 44 is found in all 59 examined species of the genus *Myotis* and 25 other vespertilionid species (e.g., Volleth and Heller [141]). Comparison of G-banded karyotypes showed that Robertsonian translocation is the prevailing mode of chromosomal change in this family. Conservation of whole chromosome arms is also evident by constancy in fundamental number in the majority of genera. Detailed analyses of G-banded chromosomal sets, however, revealed that small inversions had happened during chromosomal evolution on 7 autosomes and the X chromosome. These changes served as characters to assign the *Pipstrellus*-like genera to the tribes Vespertilionini and Pipistrellini [141]. The phylogenetic relationships inferred from this analysis were confirmed by molecular data [17]. Within Vespertilionidae, intrageneric chromosomal evolution varies from conservative to extreme. On one side there are genera with only minor chromosomal changes between species, e.g., *Myotis*, *Murina* and *Eptesicus* [35,65,143]. However, there are also genera displaying a high rate of karyotypical rearrangements as for instance *Neoromicia*, *Rhogeessa* and *Kerivoula* [142,144,145]. In the last category of highly rearranged karyotypes, FISH analyses with human or *Myotis* whole chromosome painting probes are very helpful in identification of chromosomal arms, as has been shown for *Glauconycteris* which has a reduced diploid number of 22 [56].

Despite historically recognized within Vespertilionidae, molecular and cytogenetic differences (2n = 50) [67] have recently provided support to place *Cistugo* in a distinct family [146]. Nevertheless, all members of Vespertilionoidea are characterized by conjunction of two formerly acrocentric chromosomes (7i, 7ii) into a single chromosome homologous to *Myotis* MMY7 [112]. This element is bi-armed in Molossidae and Natalidae but acrocentric in shape in Vespertilionidae and Miniopteridae (and probably also in Cistugidae where banded analyses have not been undertaken yet).

## 5. Chromosomes as Tools to Resolve Phylogenetic Problems

At a first glance, the use of chromosome characters as phylogenetic markers seems non-informative for bats. A closer look, however, has shown that many informative characters can be obtained from karyotypic work. As with other markers, the applicability depends on the taxonomic level to be investigated. For example, so far, six syntenic associations were synapomorphic for Chiroptera and Volleth and Eick [147] have discussed the limited number of investigated species has hampered the establishment of the ancestral chiropteran karyotype. Therefore, hopefully we will be headed towards a supported relationship as more syntenic associations are uncovered by chromosome painting data. At this taxonomic level (relationship to other mammalian orders), however, few characters can be reliably used [68,148] and the rapid bat radiation might be responsible for incomplete lineage sorting problems even after more robust analyses come into place. Nevertheless, karyotypic data has aided in taxonomic studies, including diagnostic characters for taxon delimitation. Thus, their importance in integrative taxonomy should not be underestimated. For example, chromosome synapomorphies gave support to the recognition of the bat familiy Cistugidae [67]. Similarly, within families with moderate to considerable chromosomal variation, relationships have been successfully delimited with the aid of chromosomal data. Examples are the subfamily Desmodontinae (Phyllostomidae), as well as the vespertilionid tribes Pipistrellini and Vespertilionini [141].

Most often, species within genera present similar karyotypical constitutions. Because of that, in several cases chromosomal data have aided solving controversial relationships. In addition, karyotypes can be easily used to reinforce proposed taxonomic changes: examples are the split of former *Tonatia* into extant *Tonatia* and *Lophostoma* [41,83,149], as well as the recognition of a new genus, *Hsunycteris* (variable 2n, including polymorphisms), distinct from other Lonchophyllinae (clade with invariable 2n = 28 FN = 50 karyotype) [150]. Finally, chromosomal data has been more successful in determining the specific status than any other taxonomic category. This is expected, since disparity of diploid numbers alone can serve as strong reproductive barriers to morphologically cryptic related populations and groups.

## 6. Conclusions

Bat genome architecture is still under-investigated. Less than 6% of the currently described species have been investigated for chromosome homology as revealed by DNA homology-based analyses, such as chromosome painting. These results showed that Robertsonian translocation is certainly the prevailing mode of chromosomal evolution in bats. However, it should be considered that it is the most easily detectable type of rearrangement. Conservation of chromosomal arm architecture is also facilitated by rarely described whole arm reciprocal translocations (WARTs) [100]. Further reduction of the diploid numbers in all-metacentric karyotypes is due to tandem fusions. In chiropteran karyotypes, there are few but impressive examples where multiple tandem fusions have been detected by FISH analyses, e.g., in the pyllostomids *Carollia brevicauda*, *Anoura cultrata*, and *Tonatia bidens* [41,59,61,96]. Inversions, which are not easily detectable by G-banding analyses alone, are less prone to convergent events than Robertsonian translocations. They are thus valuable characters for judging phylogenetic relationships. For example, a small paracentric inversion in the MMY2 homologous element has been shown to be a synapomorphic character for Pteropodiformes [110]. Given the wide range of diploid numbers and modes of chromosomal change cross lineages, bats are really fascinating creatures, not only for their wide range of ecological adaptations but also for their complexity in chromosomal evolution.

## Figures and Tables

**Table 1:** Bat families and number of cytogenetically characterized species.

Family	2n	N	Homogen	G-Banded	Zoo-FISH	References
Cistugidae	50	2	2	-	-	[67]
Craseonycteridae	nk	1	-	-	-	-
Emballonuridae	22–48	53	20	11	1	[68,69,70,71,72]
Furipteridae	34	2	1	-	-	[73]
Hipposideridae	24–52	81	27	11	7	[62,74,75,76,77]
Megadermatidae	38–54	6	3	2	1	[57,68,78]
Miniopteridae	46	25	10	3	2	[79,80,81]
Molossidae	34–52	110	50	11	3	[62,68,80]
Mormoopidae	38	15	5	8	-	[82,83]
Mystacinidae	36	2	1	-	-	[84]
Myzopodidae	26	2	1	1	1	[80]
Natalidae	36	8	2	-	-	[85,86]
Noctilionidae	34	2	2	2	-	[86,87,88]
Nycteridae	34–42	16	6	-	-	[89]
Phyllostomidae	14–46	204	106	74	25	[41,59,60,61,63,87,90,91,92,93,94,95,96]
Pteropodidae	24–58	192	45	13	4	[62,68,74,75,97]
Rhinolophidae	28–62	96	48	20	13	[62,74,75,79,97,98,99,100,101,102]
Rhinonycteridae	36–40	9	2	-	-	[67,103]
Rhinopomatidae	36–42	4	3	2	-	[104,105]
Thyropteridae	32–40	5	2	1	-	[87]
Vespertilionidae	18–52	436	189	71	12	[62,75,79,80,106]
Total	14–62	1271	525	230	69	

2n: range of diploid chromosome numbers reported; N: number of described species according to Simmons [1]; and updated by Solari and Martínez-Arias [2] for neotropical families Noctilionidae, Mormoopidae, and Phyllostomidae; plus reports from Amador et al. [3] and citations therein; Homogen: number of species with homogeneously (conventional) stained karyotypes; G-banded: number of species with G-banded karyotypes; fluorescence in situ hybridization (FISH): number of species with application of whole chromosome painting probes. References are given preferably from FISH data. Publications dealing with non-differentially stained karyotypes are mentioned only if other data are not available.

**Table 2:** Chromosomal arm combinations in Rhinolophidae and related species.

Species 2n	RHI	RPE	RSI	RRO	RSE	RMO	RLU	RLA	RFO	AST	HLA	ESP
	54	44	36	56	28	32	32	32	52	30	32	36
MMY homology												
1/3					o		x					
1/11			**x**					**x**				
1/14						**x**						**x**
1/16-17		**x**								**x**		
1/18											x	
1/20 *					x							
2/5							**x**	**x**				
2/6		**x**	**x**									
2/8ii					x							
2/9											x	
2/19						x						
2/25										x		
3/6								x				
3/7ii	x											
3/8i				**x**	**o**							
3/9												x
3/11											x	
3/13			**x**			**x**						
3/15		**x**								**x**		
4/5	**x**	**x**			**x**						**x**	
4/8ii			**x**			**x**	**x**	**x**				
4/14					x							
4/18										x		
4/19												x
5/6										x		
5/9					x							
5/10i									x			
5/1			x									
5/18						x						
6/11							**x**					**x**
6/15						**x**					**x**	
6/25					x							
7i/7ii						x						
7i/12ii				**x**			**x**	**x**				
7i/18			x									
7i/19										**x**	**x**	
7i/22i					x							
7ii/9										x		
7ii/15												x
7ii/19			**x**				**x**	**x**				
7ii/21											x	
7ii/23					x							
8i/12ii											x	
8i/20 *					o							
8i/22i						**x**	**x**	**x**	**x**			
8i/24										x		
8ii/11		x										
8ii/14										**x**	**x**	
8ii/22ii									x			
9/11						x						
9/14					x							
9/15			**x**				**x**	**x**				
9/19		x										
10i/12i					x							
10i/12ii		x										
10i/16-17						x						
10i/22ii							**x**	**x**				
10i/24			**x**	**x**								
10ii/12ii			x									
10ii/18		x										
10ii/21						**x**	**x**	**x**				
10ii/22ii					o							
11/15					x							
11/20i + ii										x		
12i/16-17			x									
12i/25						**x**	**x**	**x**	**x**		**x**	
12ii/20 *					x							
12ii/22ii						x						
13/14		x										
13/16-17							**x**	**x**				
13/18					x							
13/23										**x**	**x**	**x**
14/18							**x**	**x**				
14/23									x			
16-17/21					x							
16-17/24											**x**	**x**
18/21												x
19/24					x							
20i/22ii			x									
20i/24						**x**	**x**	**x**				
20ii/23						**x**	**x**	**x**				
20ii/25			x									
21/22i + ii										x		
22ii/24					o							

Arm combinations in Robertsonian fusion chromosomes: x single, **x** (bold) multiple occurrence; Neighboring segments due to tandem fusions in *R. sedulus*: o; i/ii proximal/distal part of respective MMY chromosome; 20 * undetermined part (either i or ii); Abbreviations: AST *Aselliscus stoliczkanus*; HLA *Hipposideros larvatus* (Hipposideridae); ESP *Eonycteris spelaea* (Pteropodidae); MMY *Myotis myotis*; RHI *R. hipposideros*; RPE *R. pearsoni*; RSI *R. sinicus*; RRO *R. rouxi*; RSE *R. sedulus*; RMO *R. morio*; RLU *R. luctoides*; RLA *R. lanosus*; RFO *R. formosae*. Arm combinations in *R. trifoliatus* equal those of *R. morio*. Data extracted from literature [62,74,75,99,100,101,102].
